# Epidemic Situation of Brucellosis in Jinzhou City of China and Prediction Using the ARIMA Model

**DOI:** 10.1155/2019/1429462

**Published:** 2019-06-13

**Authors:** Lulu Wang, Chen Liang, Wei Wu, Shengwen Wu, Jinghua Yang, Xiaobo Lu, Yuan Cai, Cuihong Jin

**Affiliations:** ^1^Department of Toxicology, School of Public Health, China Medical University, Shenyang 110122, China; ^2^Jinzhou Centre for Disease Control and Prevention, No. 8-35, Section I, Jiefang Road, Jinzhou 121000, China; ^3^Department of Epidemiology, School of Public Health, China Medical University, Shenyang 110122, China

## Abstract

**Objective:**

This study aimed to investigate the specific epidemiological characteristics and epidemic situation of brucellosis in Jinzhou City of China so as to establish a suitable prediction model potentially applied as a decision-supportive tool for reasonably assigning health interventions and health delivery.

**Methods:**

Monthly morbidity data from 2004 to 2013 were selected to construct the autoregressive integrated moving average (ARIMA) model using SPSS 13.0 software. Moreover, stability analysis and sequence tranquilization, model recognition, parameter test, and model diagnostic were also carried out. Finally, the fitting and prediction accuracy of the ARIMA model were evaluated using the monthly morbidity data in 2014.

**Results:**

A total of 3078 cases affected by brucellosis were reported from January 1998 to December 2015 in Jinzhou City. The incidence of brucellosis had shown a fluctuating growth gradually. Moreover, the ARIMA(1,1,1)(0,1,1)_12_ model was finally selected among quite a few plausible ARIMA models based upon the parameter test, correlation analysis, and Box–Ljung test. Notably, the incidence from 2005 to 2014 forecasted using this ARIMA model fitted well with the actual incidence data. Notably, the actual morbidity in 2014 fell within the scope of 95% confidence limit of values predicted by the ARIMA(1,1,1)(0,1,1)_12_ model, with the absolute error between the predicted and the actual values in 2014 ranging from 0.02 to 0.74. Meanwhile, the MAPE was 19.83%.

**Conclusion:**

It is suitable to predict the incidence of brucellosis in Jinzhou City of China using the ARIMA(1,1,1)(0,1,1)_12_ model.

## 1. Introduction

Brucellosis is an infective and allergic anthropozoonosis caused by *Brucella*. According to the *Law of the People's Republic of China on the Prevention and Treatment of Infectious Diseases*, brucellosis is a natural focus infectious disease listed as a class B infectious disease. Meanwhile, it is also a class B animal epidemic disease according to *International Office of Epizootics* (OIE). Patients infected with *Brucella* show the main symptoms such as fever, headache, fatigue, hidrosis, neuralgia, and bone, joint, and muscle pain [1]. The incubation period fluctuates greatly, which ranges from 1 to 3 weeks. Generally, human beings are not the source of infection, but they are vulnerable to most bacteria of *Brucella* genus [2,3]. In China, sheep account for the main source of *Brucella*.

Brucellosis has spread all over the world, especially in developing areas such as Asia, Central and South America, and Mediterranean region [3–5], but the regulation of brucellosis started late at home and abroad. In order to keep abreast of the epidemic situation of brucellosis, China has set up monitoring stations in 14 provinces (districts) since 1990. The epidemic situation of brucellosis in Liaoning Province ranks the 5^th^ in China, where 14 cities are afflicted with the prevalence of brucellosis at various degrees. Moreover, most cases suffer from *Brucella melitensis* [1].

In recent years, the incidence of brucellosis in Jinzhou City has been on the rise. Notably, the incidence of brucellosis in Jinzhou took the first place in Liaoning Province from 2005 to 2008. One of the purposes of monitoring is to predict, while the existing studies only monitor without prediction. Accordingly, the research in this field is almost blank in Jinzhou, and it is difficult for doctors to grasp the incidence trend. It is of great significance to analyze the monitoring data of brucellosis by mathematical model methods and predict the epidemic dynamics to better grasp the epidemic trend of brucellosis in Jinzhou City. In addition, an actual value obviously exceeding the 95% confidence limit of predictive values warns a possible outbreak of infectious diseases [6]. In this way, targeted measures for disease prevention and control would be carried out based on the predicted data. Thus, we can efficiently control the epidemic of infectious diseases.

ARIMA is often adopted in the short-term prediction of infectious diseases, which can be ascribed to its virtues of compatibility to complicated factors, simple model structure, easy operation, economy, and practicability. ARIMA is applicable to both stationary and nonstationary time series. Therefore, it has been widely applied in predicting seasonal and periodic infection. For instance, Lee had predicted the morbidity of human and bovine brucellosis with time-series analysis in South Korea [7]. In China, Bai [8] and Yang [9] had predicted the epidemic situation of human brucellosis in both Shanxi Province and Shandong Province, respectively. Nevertheless, few reports are available so far regarding human brucellosis in other districts of China.

In the current study, the incidence characteristics of brucellosis within the past two decades were described. For the first time, the ARIMA model was established to predict the incidence trend of brucellosis in Jinzhou. Hopefully, the current study could provide recent epidemic characteristics of brucellosis in Jinzhou City of Northeast China, establish scientific basis for the prevention and treatment strategies of brucellosis, and offer clues for its management and warning.

## 2. Methods

### 2.1. Data Sources

All data of human brucellosis were collected from the China Information System for Disease Control and Prevention. All cases had been laboratory confirmed.

### 2.2. Description of Epidemic Characteristics

The incidence data of brucellosis from 1998 to 2015 were classified into annual and monthly cases for subsequent analyses. Later, the monthly incidence data, which served as the basic data for the time-series model, were calculated according to the total population in the region. Moreover, statistical charts were used to describe the incidence trend and epidemic characteristics.

### 2.3. ARIMA Model

The monthly morbidity data from 2004 to 2013 were adopted to establish the ARIMA model and then to predict brucellosis morbidity from 2005 to 2014 to identify its stationarity and availability.

Autoregressive integrated moving average (ARIMA) is a time-series model, in which the orders of autoregressive and moving average parts are “p” and “q” denoted by AR(p) and MA(q), respectively. The time series in the ARIMA model should be a stationary and stochastic sequence with zero mean. As a result, the nonsmooth sequence should be converted into stationary series by difference transformation so that the ARMA model becomes the ARIMA model. Specifically, if “d” indicates the difference order, the model is written as ARIMA(p, d, q) without seasonal component, ARIMA(sp, sd, sq) with seasonal components, and ARIMA(p, d, q)(sp, sd, sq) complex model. The complex model, which is suitable for a general sequence, is the most advantageous among these models. Consequently, it is necessary to order p, d, q, sp, sd, and sq so as to construct the ARIMA model. Generally, the successive steps were processed to construct the ARIMA model including stationarity, identification, and estimation, as well as diagnostic and forecasting.

In this study, the monthly incidence data of brucellosis from 2004 to 2013 were selected in consideration of their integrity and stationary trend to the model. Subsequently, the morbidity of brucellosis from 2005 to 2014 was predicted, and the predictive accuracy of the ARIMA model was finally estimated using the monthly morbidity data in 2014.

#### 2.3.1. Sequence Stationarity

The time sequence (monthly incidence data of brucellosis from 2004 to 2013) was found to be nonstationary. Therefore, the methods of square-root transformation, once common difference, and once seasonal difference had been conducted successively to transform this nonsmooth sequence into a stationary one. Subsequently, the primitive sequence diagram and the transformed sequence diagram were used to evaluate the stationarity and trend. Moreover, the sequence stationarity was tested by the augmented Dickey–Fuller (ADF) test using the EViews 6.0 software.

#### 2.3.2. Identification

Firstly, the randomness, stationarity, and seasonal characteristics of the time sequence were recognized and analyzed through observing the autocorrelation function (ACF) and partial autocorrelation function (PACF). Afterwards, orders of the model were generally determined from 0 to 2 in accordance with the AIC and BIC, whose orders were rarely more than 2. Several rough models had been recognized by differently combining 0, 1, and 2; meanwhile, the optimal model with minimum AIC and BIC was selected finally.

#### 2.3.3. Estimation and Diagnosis

The appropriateness of the candidate model was diagnosed using the error sequence “*e*_*t*_-test”, where “*e*_*t*_” was named the residual error, indicating the *D* value between the actual and predicted morbidity. It was required that the residual error should be white noise for an appropriate model. The white noise of the residual series was recognized using the Box–Ljung test. In other words, the residual error must be random with no statistical significance in the residual correlation test. According to the residual irrelevant principle [10], the model was suitable for forecasting if its residual series was white noise; otherwise, the model should be improved and identified again [11,12].

#### 2.3.4. Forecasting and Assessment

The optimal ARIMA model was adopted to predict the monthly morbidity data from 2005 to 2014, the effect and accuracy of which were subsequently assessed with 2 methods. On the one hand, the fitting effect of the ARIMA model between the actual and the predicted values was determined by observing whether the actual values had fallen within the scope of 95% confidence limit of the predicted values. On the other hand, the mean absolute percentage error (MAPE) was calculated to evaluate the accuracy of the ARIMA model.

### 2.4. Statistical Analysis

The epidemic characteristics of brucellosis were described using Excel software (17.0).

Meanwhile, SPSS (13.0) software was used to analyze the time series, define the time variable, and estimate the stationarity. Moreover, the sequence and correlation were plotted, and the Box–Ljung test was conducted. ARIMA model fitting tests were also carried out, including standard error, log-likelihood, AIC and BIC, and residual variance analysis. Besides, model diagnosis was performed, including parameter *t*-test, correlation test, and Box–Ljung test for “*e*_*t*_.” Finally, the predicting effect of the ARIMA model was determined using the confidence limit along with MAPE.

In addition, the stationarity of the time series was inspected by the ADF test using EViews 6.0 software.

## 3. Results

### 3.1. Epidemiological Characteristics of Brucellosis in Jinzhou from 1998 to 2015

#### 3.1.1. Overall Distribution

**Figure 1:**
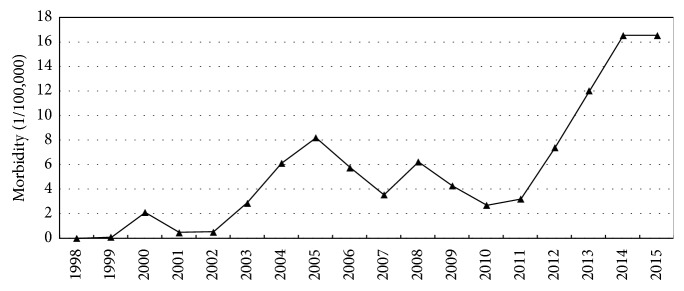
Brucellosis morbidity in Jinzhou from 1998 to 2015. The incidence had a wave-like increasing tendency year by year with the trend of rectilinear rise after 2011. The morbidity was 16.53 per 100,000 which was the peak incidence since 1998.

Firstly, the overall distribution data of brucellosis from 1998 to 2015 were collected and analyzed. As is shown in Figure 1, a total of 3078 brucellosis cases were observed from 1998 to 2015. The incidence showed a wave-like increasing tendency year by year, with the trend of rectilinear rise after 2011. 513 cases were reported in 2014 and 2015, respectively, and the morbidity was 16.53 per 100,000, which stood for the peak incidence since 1998.

#### 3.1.2. Time Distribution

**Figure 2:**
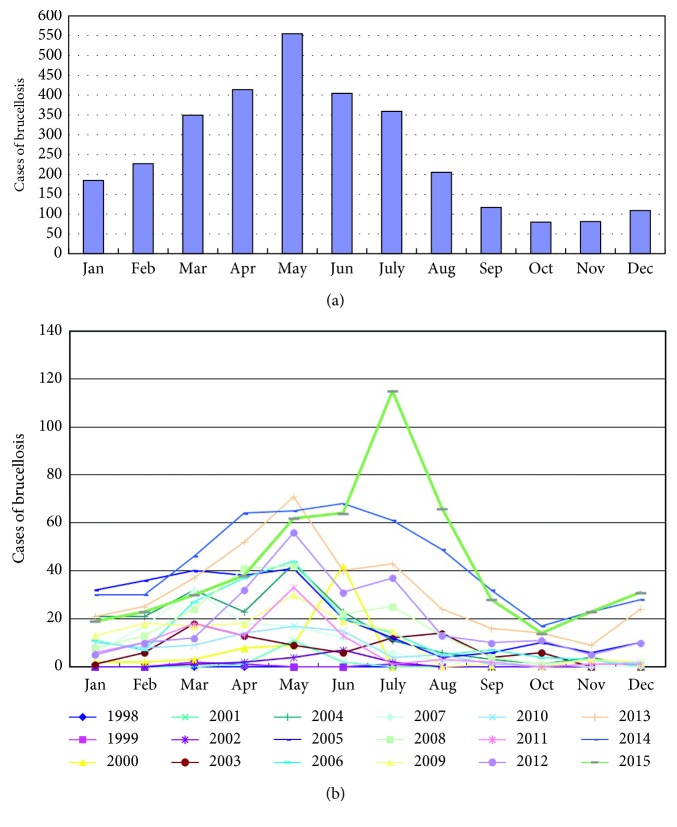
Monthly distribution and incidence trend of brucellosis in Jinzhou from 1998 to 2015. (a) Cases of brucellosis in different months of a year. There were 554 cases in May from 1998 to 2015, which was the peak period of brucellosis. The lowest incidence fell in spare time during the winter. (b) Monthly incidence trend of brucellosis each year from 1998 to 2015. The incidence trend and characteristic basically coincided in different years, except for 2015 when 115 patients were reported in July.

As is shown in Figures 2(a) and 2(b), a total of 554 cases were reported in May from 1998 to 2015, which represented the peak period of brucellosis during the whole year. Moreover, the incidence trend and characteristics basically coincided in most years, except for 2015 when 115 patients were reported in July.

### 3.2. ARIMA Model Forecasting Analysis

#### 3.2.1. Sequence Characteristic Analysis and Transformation

**Figure 3:**
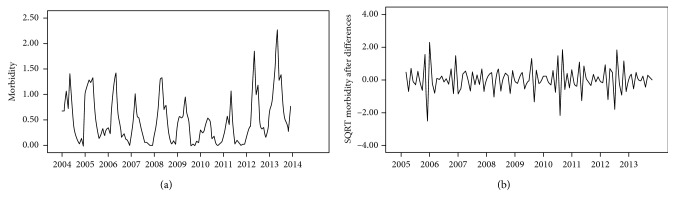
Monthly morbidity sequence and transformation of brucellosis in Jinzhou from 2004 to 2013. (a) Original time sequence. The original time sequence took up a upward or downward trend with a seasonal cycle rule, which was not smooth and had unequal variances. (b) Transformed time sequence. The original sequence was transformed into a random one by square-root transformation, once common difference, and once seasonal difference successively. ADF test: *t* = −8.66 and *P* < 0.01; the trend of time sequence turned random and stationary.

Firstly, a monthly sequence from 2004 to 2013 was calculated and its chart was drawn, as shown in Figure 3(a). The original sequence showed an upward or downward trend with a seasonal cycle rhythm, which was not smooth and had uneven variances. Therefore, the original sequence was transformed into a random one through the methods of square-root transformation, once common difference, and once seasonal difference successively. After that, the time sequence displayed a random and stationary trend (Figure 3(b); ADF test: *t* = −8.66 and *P* < 0.01).

#### 3.2.2. Identification

**Figure 4:**
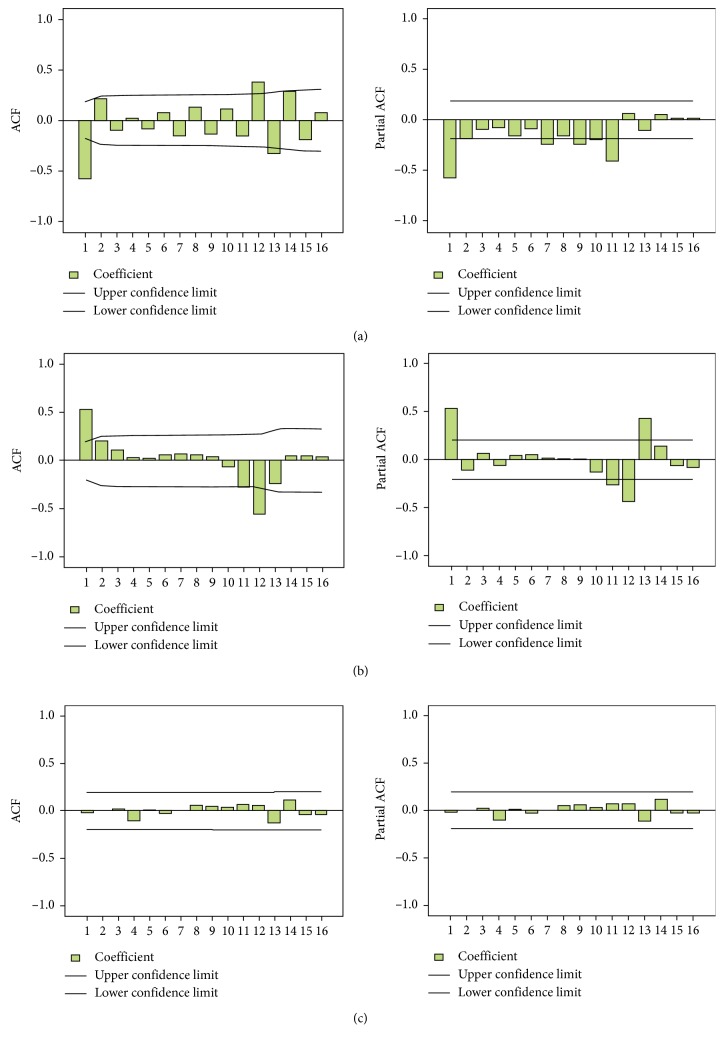
ACF and PACF charts after twice differences and the residual series of the ARIMA(1,1,1)(0,1,1)_12_ model. (a) Once common difference ACF and PACF charts. In the once common difference ACF chart, when lag = 1, the autocorrelation coefficient broke through the confidence interval. In the once common difference PACF chart, when lag = 1, the partial autocorrelation coefficient broke through the confidence interval apparently, while the coefficient hardly broke through the confidence limit when lag = 2. (b) Once seasonal difference ACF and PACF charts. In the once seasonal difference PACF chart, the partial autocorrelation coefficient broke through the limitation apparently when lag = 1. (c) The residual error correlation, ACF, and PACF of the ARIMA(1,1,1)(0,1,1)_12_ model. This model fell in the confidence limit, and there was no obvious correlation.

The order of the model was determined according to ACF (Figure 4(a)) and PACF (Figure 4(b)) after once common difference and once seasonal difference. ACF was the related coefficient between the prior and lag sequences. The autocorrelation coefficient would decline exponentially or in a sinusoidal wave and would approach zero when lag > q. As shown in the ACF chart (Figures 4(a) and 4(b)), when lag = 1, the autocorrelation coefficient would break through the confidence interval, which shows stronger relevance within 1 order. Therefore, q = 1 and sq = 0 or 1 were preliminarily identified.

Previous values which were also the AR(p) order were needed to forecast an actual value. It was shown in the once common difference PACF chart (Figure 4(a)) that the partial autocorrelation coefficient apparently broke through the confidence limit when lag = 1, while the coefficient hardly broke through the confidence limit when lag = 2. Consequently, 2 orders were enough for modeling. In addition, it was illustrated in the once seasonal difference PACF chart (Figure 4(b)) that the partial autocorrelation coefficient outstandingly broke through the confidence limit when lag = 1. As a result, p = 1 or 2 and sp = 0 or 1 were preliminarily ascertained.

After the orders were identified, we obtained 8 rough models: ARIMA(1,1,1)(0,1,0)_12_, ARIMA(1,1,1)(0,1,1)_12_, ARIMA(1,1,1)(1,1,0)_12_, ARIMA(1,1,1)(1,1,1)_12_, ARIMA(2,1,1)(0,1,0)_12_, ARIMA(2,1,1)(0,1,1)_12_, ARIMA(2,1,1)(1,1,0)_12_, and ARIMA(2,1,1)(1,1,1)_12_.

#### 3.2.3. Estimation and Diagnosis of Model

Parameter estimation and correlation analysis of 8 rough models are presented in Table 1.

According to the results of correlation analysis (Table 2), no correlation was observed among parameters of three candidate models. As a result, these three models were all accepted.

Furthermore, as shown in Table 3, there were no statistically significant differences in ARIMA(1,1,1)(0,1,1)_12_ and ARIMA(1,1,1)(1,1,0)_12_ models (*P* > 0.05). In other words, no obvious correlation was observed and the residual series was white noise.

Subsequently, the goodness of fit of three models (Table 4) was analyzed. Both the AIC and BIC values of the ARIMA(1,1,1)(0,1,1)_12_ model were found to be minimum, which meets the selection criterion. Moreover, the residual series of the ARIMA(1,1,1)(0,1,1)_12_ model was also white noise (Figure 4(c)). Therefore, the ARIMA(1,1,1)(0,1,1)_12_ model was the optimal model for prediction.

#### 3.2.4. Forecasting Using ARIMA Model

**Figure 5:**
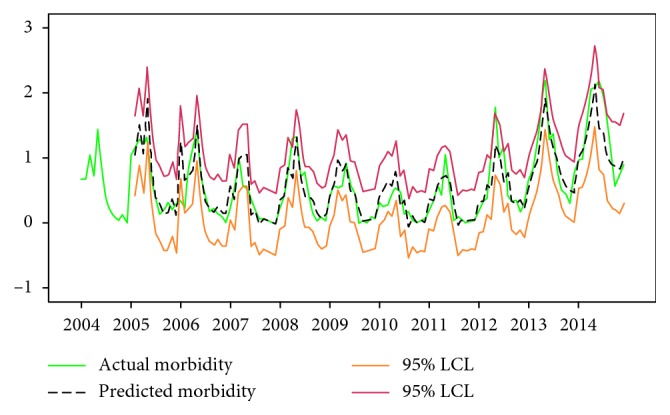
Fitting status situation between actual and predicted morbidity from 2005 to 2014. Red and orange lines indicate the upper and lower scope of 95% confidence limit of predicted incidence of brucellosis, while a green line indicates the actual incidence values and a black dotted line indicates the predicted incidence.

As shown in Figure 5, the monthly morbidity data from 2005 to 2014 were predicted using the ARIMA(1,1,1)(0,1,1)_12_ model based on the morbidity of brucellosis from 2004 to 2013, the results of which suggested that the predicted values fitted well with the actual values. Notably, the actual values in 2014 fell in the 95% confidence limit of the ARIMA(1,1,1)(0,1,1)_12_ model, and the MAPE was 19.83% (Table 5).

## 4. Discussion

In Jinzhou City, there had been no case of brucellosis until the year of 1983, when domestic brucellosis outbreak among humans was first discovered. Since then, brucellosis cases have been reported every year and on an uptrend. The rapid development of animal husbandry as well as the sharp increases in herdsmen and butchers has made great contributions to the above phenomenon. Moreover, the convenient transportation facilitating regional dealings, together with the expansive circulation of livestock, has given rise to the extensive spread of *Brucella* between the infected and healthy animals [13, 14]. Last but not least, *Brucella* can be more easily brought in and spread locally due to the neglected animal immunity and the lack of vaccine in many villages and towns.

Brucellosis displays an obvious seasonality, with spring being the epidemic season [15]. In this study, high incidence occurred from spring to autumn during the busiest period for farmers, while the lowest incidence was observed in winter when the farmers had spare time. In time distribution, the incidence in July 2015 was the highest during the same period of all time, and the reason is that there were three outbreaks in July 2015.

Time-series analysis is a method to extrapolate predictions, in which a mathematical model is established according to the regularity and trend of the historical observed values with time. In this study, the prevalence data of livestock, instability of the animal brucellosis rule, and complexity of environment factors [16] can hardly be obtained, which limits the prediction of brucellosis. Nonetheless, time-series analysis can overcome these major obstacles. Time has taken the place of other influencing factors in many ways, such as trend, season, and human factors [17], regardless of the causality between variables. Time-series analysis, including the ARIMA model, has been widely used in predicting infectious diseases in recent years. For instance, some scholars have used this model to predict morbidity of TB, mumps, measles, encephalitis B, and hand-foot-and-mouth disease [18–22]. These diseases are similar to brucellosis in time distribution. The ARIMA model has taken the time-series trend, aspection, and interference into consideration, which can also quantify the expression by virtue of model parameters [23, 24]. The incidence sequence of brucellosis in Jinzhou has met the requirement of the time series; therefore, historical data within the past 10 years are selected in this study. Such time series is long enough and relatively steady, which is suitable and practicable for forecasting the incidence of brucellosis using the ARIMA model. It is possible to be applied in predicting the incidence of human brucellosis.

However, there are some potential differences between the predicted and actual values due to the complicated related factors of brucellosis. As a result, the parameters should be identified and modified repeatedly so as to pick out the optimal model with the best fitting degree. In this study, ARIMA(1,1,1)(0,1,1)_12_ was confirmed to be the optimal model. Almost all the actual morbidity data in 2014 had fallen within the range of 95% confidence interval and MAPE was as low as 19.83%, indicating an ideal forecasting effect. The actual morbidity fluctuating within the 95% confidence limit indicates the normal incidence, while the epidemic situation would be sharply different from the former epidemic pattern if the actual values break through such a range. Therefore, we must be alert of the possibilities of infectious disease outbreak [1], and relevant personnel should adopt specific corresponding measures ahead of time to control and treat the secondary widespread brucellosis.

Nevertheless, the ARIMA model is not a fixed pattern; therefore, various strategies should be adopted in accordance with a specific situation. Existing studies have indicated that the ARIMA model is more applicable for short-term (less than one year) prediction, which may be attributed to various influencing factors of infection as well as the complicated interactions among them. However, ARIMA is also potentially suitable for long-term prediction under the premise of a relatively stable incidence trend and external factors, just like our findings that ARIMA(1,1,1)(0,1,1)_12_ is a good prediction model for brucellosis in a certain district. Furthermore, the incidence of brucellosis may be remarkably different due to the restriction of geographical conditions in different areas. In this study, the ARIMA(1,1,1)(0,1,1)_12_ model is more suitable for predicting the incidence in Jinzhou, which should be further amended more or less when applied to other districts.

Like other studies, the ARIMA model is also associated with certain limitations. For instance, it proposes the linear relation within time-series data by taking the time factor into consideration only, rather than pathogen, host, social economy, and natural environment factors [7]. Accordingly, all the other influencing factors should be further clarified in future monitoring and taken into account when taking preventive measures in practical works.

Such a model provides important theoretical basis and clues for the early warning and intervention of infectious diseases. On the basis of this study, we can further carry out brucellosis research in combination with relevant departments and comprehensively analyze its etiology, incidence trend and distribution, influencing factors, and so on. Brucellosis prevention is a long-term and hard task, which requires cooperation of various departments, such as the department of health and the department of animal husbandry. It is of great necessity for the government at all levels to strengthen the management of livestock market and implement strict quarantine. Moreover, the surveillance of the epidemic situation between humans and animals, the health education, the propaganda of the knowledge for brucellosis prevention, the early detection, and the timely treatment should also be enhanced during strictly managing livestock quarantine transaction and eliminating infected animals. In this way, the epidemic of brucellosis in Jinzhou and even other areas can be effectively prevented and controlled.

## 5. Conclusion

The incidence of brucellosis from 1998 to 2015 had shown a fluctuating growth gradually in Jinzhou, which peaked in 2014 and 2015. The ARIMA models have been successfully established, among which ARIMA(1,1,1)(0,1,1)_12_ is suitable for predicting the incidence of brucellosis in Jinzhou.

## Figures and Tables

**Table 1:** Parameter estimation of 8 candidate models.

	*B*	SE	*t*	*P*
ARIMA(1,1,1)(0,1,0)_12_				
AR1	0.59	0.09	6.92	0.00
MA1	0.99	0.17	5.91	0.00
ARIMA(1,1,1)(0,1,1)_12_				
AR1	0.55	0.11	5.16	0.00
MA1	0.93	0.06	15.59	0.00
SMA1	0.71	0.12	5.99	0.00
ARIMA(1,1,1)(1,1,0)_12_				
AR1	0.54	0.11	4.86	0.00
MA1	0.92	0.06	15.71	0.00
SAR1	−0.45	0.09	−5.14	0.00
ARIMA(1,1,1)(1,1,1)_12_				
AR1	0.55	0.11	5.21	0.00
MA1	0.93	0.06	15.66	0.00
SAR1	0.09	0.18	0.48	0.63
SMA1	0.78	0.22	3.63	0.00
ARIMA(2,1,1)(0,1,0)_12_				
AR1	0.61	0.10	6.11	0.00
AR2	−0.02	0.10	−0.15	0.88
MA1	0.99	0.15	6.86	0.00
ARIMA(2,1,1)(0,1,1)_12_				
AR1	0.55	0.11	5.00	0.00
AR2	0.00	0.11	−0.01	0.99
MA1	0.93	0.07	14.13	0.00
SMA1	0.71	0.12	5.95	0.00
ARIMA(2,1,1)(1,1,0)_12_				
AR1	0.54	0.11	4.76	0.00
AR2	0.02	0.11	0.22	0.83
MA1	0.93	0.06	14.90	0.00
SAR1	−0.45	0.09	−5.14	0.00
ARIMA(2,1,1)(1,1,1)_12_				
AR1	0.55	0.11	5.03	0.00
AR2	−0.01	0.11	−0.08	0.94
MA1	0.93	0.07	13.98	0.00
SAR1	0.09	0.18	0.49	0.63
SMA1	0.78	0.22	3.61	0.00

*Note*. The parameter estimation of the ARIMA(1,1,1)(0,1,0)_12_, ARIMA(1,1,1)(0,1,1)_12_, and ARIMA(1,1,1)(1,1,0)_12_ models showed *P* < 0.05, but for other models, *P* > 0.05.

**Table 2:** Correlation analysis of 3 candidate models.

	Nonseasonal model		Seasonal model	
	AR1	MA1	SAR1	SMA1
ARIMA(1,1,1)(0,1,0)_12_				
AR1	1	0.34	—	—
MA1	0.34	1	—	—
ARIMA(1,1,1)(0,1,1)_12_				
AR1	1	0.64	—	0.04
MA1	0.64	1	—	−0.10
SMA1	0.04	−0.10	—	1
ARIMA(1,1,1)(1,1,0)_12_				
AR1	1	0.66	0.09	—
MA1	0.66	1	0.15	—
SAR1	0.09	0.15	1	—

*Note*. The parameters of three candidate models are all less than 1, so there is hardly any correlation.

**Table 3:** Correlation analysis of residual error of 3 candidate models.

Lag	ARIMA(1,1,1)(0,1,0)_12_			ARIMA(1,1,1)(0,1,1)_12_			ARIMA(1,1,1)(1,1,0)_12_		
	Autocorrelation	Box–Ljung value	*P*	Autocorrelation	Box–Ljung value	*P*	Autocorrelation	Box–Ljung value	*P*
1	−0.00	0.00	0.97	−0.02	0.04	0.84	−0.02	0.06	0.81
2	−0.06	0.41	0.82	0.01	0.05	0.98	0.01	0.06	0.97
3	0.06	0.76	0.86	0.02	0.08	0.99	0.07	0.69	0.88
4	−0.07	1.28	0.87	−0.10	1.24	0.87	−0.12	2.18	0.70
5	0.02	1.31	0.93	0.02	1.26	0.94	0.03	2.27	0.81
6	0.06	1.69	0.95	−0.03	1.38	0.97	0.03	2.37	0.88
7	0.02	1.75	0.97	0.00	1.38	0.99	−0.04	2.53	0.93
8	0.09	2.65	0.96	0.06	1.79	0.99	0.06	2.89	0.94
9	0.11	3.92	0.92	0.05	2.11	0.99	0.08	3.61	0.94
10	0.06	4.31	0.93	0.04	2.29	0.99	−0.02	3.66	0.96
11	0.07	4.92	0.94	0.07	2.93	0.99	0.11	5.21	0.92
12	−0.37	21.46	0.04	0.06	3.38	0.99	−0.02	5.26	0.95
13	−0.04	21.67	0.06	−0.12	5.19	0.97	−0.11	6.78	0.91
14	0.20	26.86	0.02	0.11	6.83	0.94	0.16	9.80	0.78
15	−0.00	26.86	0.03	−0.04	7.02	0.96	−0.04	9.97	0.82
16	−0.01	26.88	0.04	−0.04	7.20	0.97	−0.02	10.03	0.87

*Note*. The correlation analysis of residual error of ARIMA(1,1,1)(0,1,1)_12_ and ARIMA(1,1,1)(1,1,0)_12_ models showed that neither of them had statistical significance (*P* > 0.05), so there was no obvious correlation and residual series was white noise.

**Table 4:** Comparision of goodness of fit of 3 candidate models.

	ARIMA(1,1,1)(0,1,0)_12_	ARIMA(1,1,1)(0,1,1)_12_	ARIMA(1,1,1)(1,1,0)_12_
Log-likelihood	−12.42	1.97	−1.97
AIC	30.85	4.07	11.93
BIC	38.87	14.76	22.63

*Note*. Both the AIC and BIC values of the ARIMA(1,1,1)(0,1,1)_12_ model were minimum.

**Table 5:** Comparison between actual and predicted monthly morbidity in 2014.

Month	Actual value	Predicted value	95% confidence limit	Error absolute value	Absolute percentage error
Jan	0.97	0.99	0.53–1.46	0.02	0.02
Feb	0.97	1.10	0.55–1.65	0.13	0.13
Mar	1.48	1.33	0.74–1.92	0.15	0.10
Apr	2.06	1.62	1.00–2.23	0.44	0.21
May	2.09	2.12	1.50–2.75	0.03	0.01
Jun	2.19	1.45	0.82–2.09	0.74	0.34
July	1.97	1.41	0.76–2.05	0.56	0.28
Aug	1.58	1.02	0.36–1.67	0.56	0.35
Sep	1.03	0.90	0.23–1.56	0.13	0.13
Oct	0.55	0.88	0.20–1.55	0.33	0.60
Nov	0.74	0.82	0.14–1.50	0.08	0.11
Dec	0.90	0.99	0.31–1.69	0.09	0.10

*Note*. The error absolute value is the *D* value between actual value and predicted value. The absolute percentage error is the ratio of error absolute value and actual value. MAPE is the mean value of absolute percentage error, and the value is 19.83%.

## Data Availability

All data of human brucellosis were collected from the China Information System for Disease Control and Prevention. All cases had been laboratory confirmed.

## Abbreviations

ACF: Autocorrelation function
ADF test: Augmented Dickey–Fuller test
AIC: Akaike's information criterion
ARIMA: Autoregressive integrated moving average
AR(p): Autoregressive(p)
BIC: Bayesian information criterion
MA(q): Moving average(q)
MAPE: Mean absolute percentage error
PACF: Partial autocorrelation function
SE: Standard error
UHT: Ultraheat-treated.
